# Successful laparoscopic management of acute abdominal pain due to spontaneous rupture of subserosal vessels overlying a uterine fibroid: a case report and surgical video

**DOI:** 10.1186/s12905-022-01970-0

**Published:** 2022-09-23

**Authors:** Toshimitsu Maemura, Shigeru Fujita, Noriko Morita, Keiichi Furusawa, Kayo Mitamura, Kenji Nishizawa, Kuniaki Ota

**Affiliations:** 1 Toho University, 6-11-1 Omori-Nishi, Ota-ku, Tokyo, 143-8751 Japan; 2 Department of Obstetrics and Gynecology, Tokyo Rosai Hospital, Japan Labor Health and Safety Organization, 4-13-21 Omori-minami, Ota-ku, Tokyo, 143-0013 Japan

**Keywords:** Acute abdomen, Hemoperitoneum, Laparoscopic myomectomy, Uterine fibroid

## Abstract

**Background:**

Acute abdomen comprises several emergencies. Hemoperitoneum associated with uterine fibroids, which can present as acute abdominal pain, is rare and difficult to diagnose. Especially, spontaneous hemorrhage from the rupture of the superficial vessels overlying a uterine fibroid is extremely rare, and its diagnosis and management have not been established.

**Case presentation:**

We report a case of a 55-year-old woman who presented at our hospital with acute abdomen. After performing a computed tomography scan, we conducted a laparoscopic examination and diagnosed hemoperitoneum of ambiguous origin. We treated the patient surgically, performing a laparoscopic myomectomy to remove the origin of the hemorrhage. The patient recovered well.

**Conclusions:**

We report a case of hemoperitoneum of ambiguous origin that was diagnosed laparoscopically and treated by laparoscopic myomectomy to remove the origin of the hemorrhage. Surgeons should rapidly diagnose and manage acute abdominal pain in women with a history of uterine fibroids to prevent severe morbidity or even mortality. Therefore, laparoscopic surgery is recommended in patients with stable hemodynamics.

**Supplementary Information:**

The online version contains supplementary material available at 10.1186/s12905-022-01970-0.

## Background

Uterine fibroids are the most common benign pelvic tumors in women of reproductive age [1]. The typical symptoms are hypermenorrhea, prolonged menstrual bleeding, menorrhagia, abdominal protrusion, infertility, and recurrent pregnancy loss [2]. The management of uterine fibroids depends on the number, size, and location of the tumors and can include medical or surgical interventions [3]. Although main strategies are definitely considered as surgical intervention, medical treatments as the first-line therapy are considered to preserve fertility and avoid surgery. Especially, the newest medical treatment options, such like gonadotropin-releasing hormone (GnRH) analogs, GnRH antagonists and selective progesterone receptor modulators, for symptomatic Uterine fibroids are now developed for women with uterine fibroids [4, 5]. Myomectomy is finally selected for patient who has severe symptomatic uterine fibroids with anemia, hypermenorrhea and abdominal pain since each medical treatment has its own advantages and disadvantages and is impossible to completely disappear into the abdominal cavity.

Acute abdomen comprises several emergencies; however, surgical and medical emergencies should be differentiated. Clinical symptoms arise mainly from peritoneal irritation caused by blood effusion, and the differential diagnosis includes ectopic pregnancy, adnexal torsion, neoplasm, and pelvic inflammatory disease [6, 7]. Hemoperitoneum of gynecological origin may occur in many causal factors of various gynecological emergencies such as ectopic pregnancies and ruptured corpus luteal cysts [8]. Hemoperitoneum associated with uterine fibroids is extremely rare [9] and difficult to diagnose preoperatively.

Herein, we report the first case of hemoperitoneum associated with spontaneous hemorrhage from the rupture of the superficial vessels overlying the uterine fibroid that was diagnosed and enucleated laparoscopically, despite a preoperative suspicion of torsion of a subserosal uterine fibroid. This case is reported in accordance with the SCARE criteria [10].

## Case presentation

**Fig. 1:**
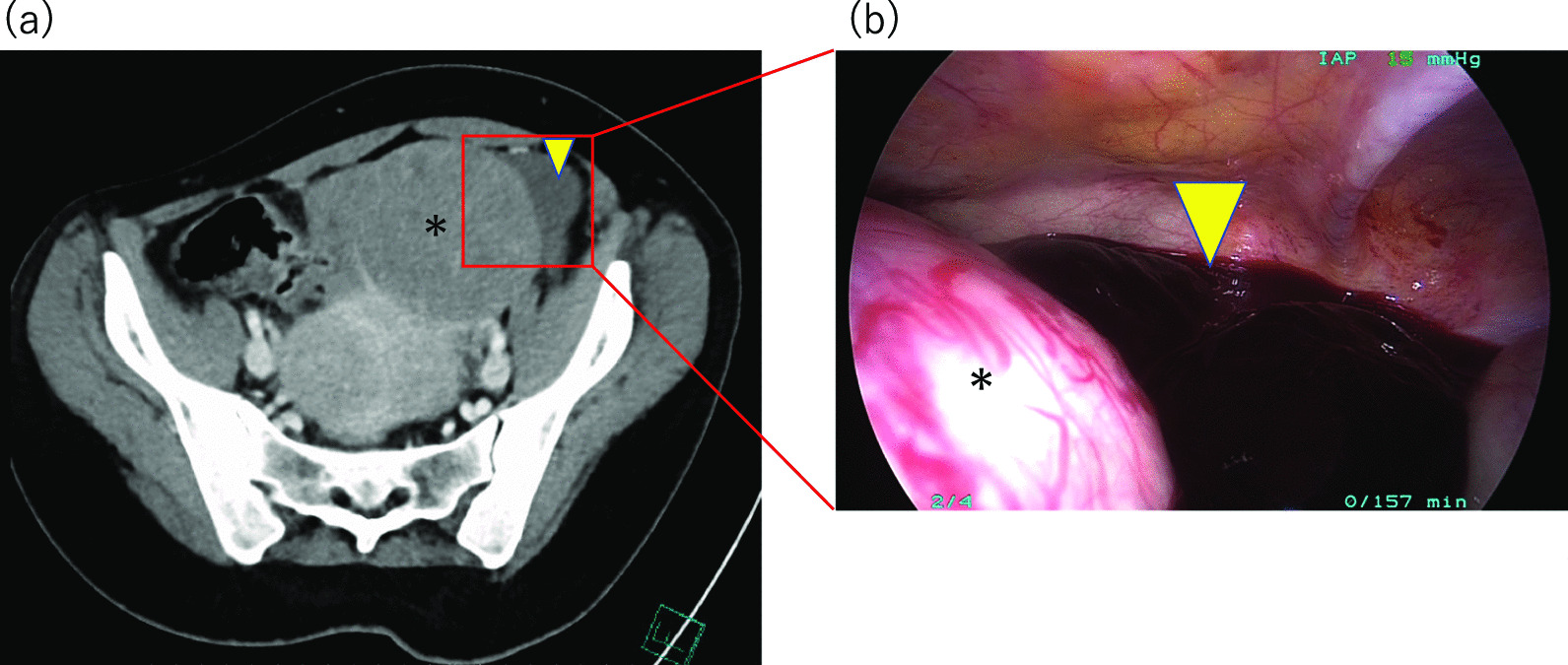
Preoperative computed tomography and intra-operative laparoscopy images identifying the hemoperitoneum. **a** Abdominal contrast-enhanced computed tomography showed a 7.9-cm fibroid (asterisk) and peritoneal fluid with an imaging density suggestive of blood (yellow arrowhead). **b** Exploratory laparoscopy identified the hemoperitoneum consistent with computed tomography (yellow arrowhead)

**Fig. 2:**
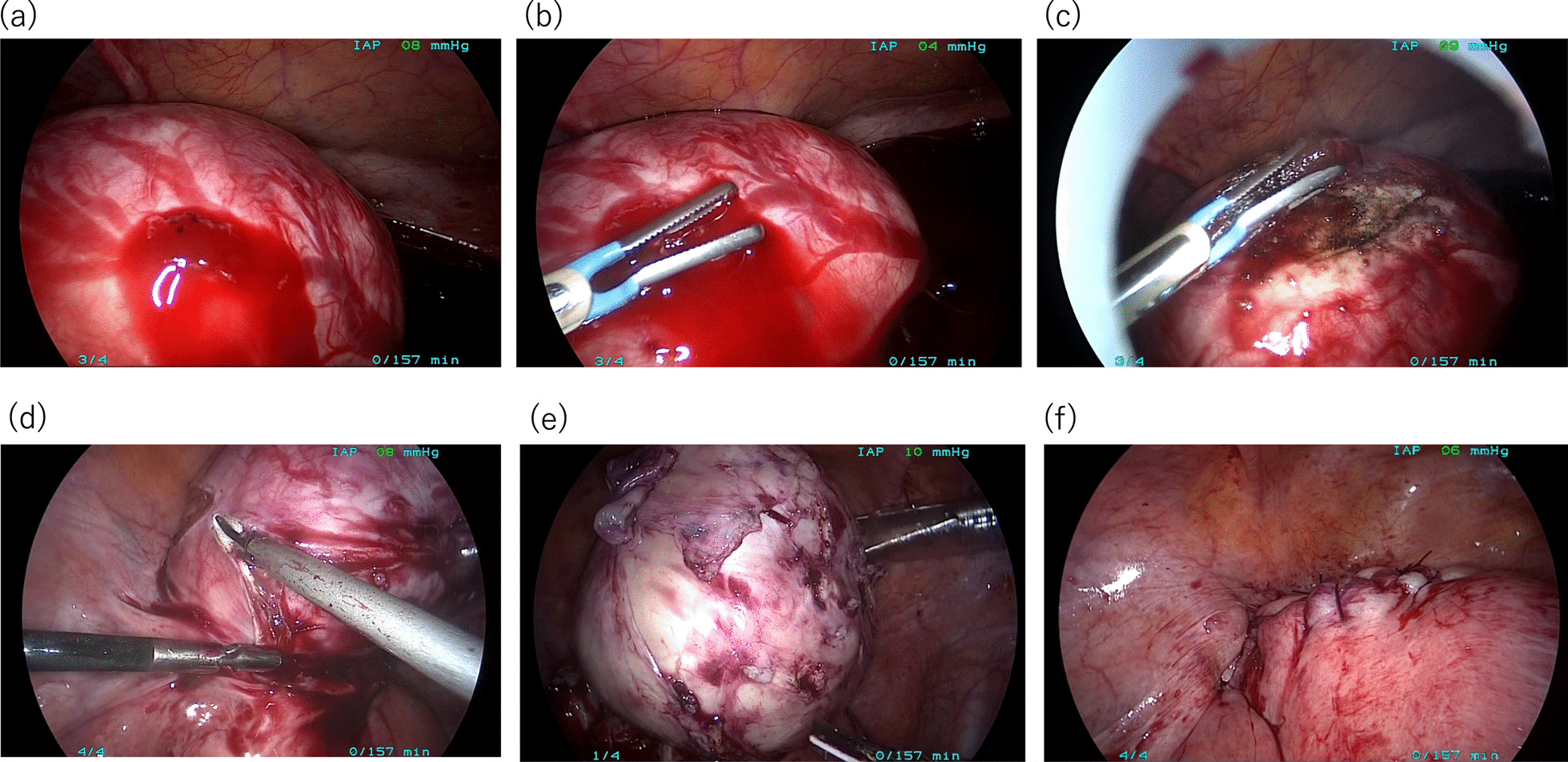
Intra-operative procedures during the laparoscopy. **a** Exploratory laparoscopy identified the bleeding site on the superficial vessels overlying the uterine fibroid. **b**, **c** Bipolar coagulation forceps were used to obtain hemostasis at the site of hemorrhage. **d** The uterus was incised with harmonic scalpels to access the uterine fibroid. **e**, **f** The uterine fibroid was laparoscopically enucleated, and the uterine defect was completely sutured for hemostasis and reconstructed in a uterine-sparing surgery

A 55-year-old gravida 2 Japanese woman presented to the emergency department with complaints of acute-onset lower abdominal pain on day 30 of her menstrual cycle. She reported a history of regular menstrual cycles with 4–5 days of bleeding each month. She had a medical history of uterine fibroids and no remarkable family history. Her heart rate, blood pressure, and temperature were 103 beats per minute, 111/81 mm Hg, and 36.8 °C, respectively. Physical examination revealed generalized tenderness and a positive Blumberg sign around the right iliac fossa. Vaginal ultrasonography revealed a pelvic mass with hyperechoic fluid filling almost the entire abdominal cavity. On admission, the total white blood cell count was 7900/μL, and the C-reactive protein level was 0.2 mg/L. The patient was transfused with four units of packed red blood cells since she had a reduced hemoglobin level (6.6 g/dL) and hematocrit (22.3%). An abdominal contrast-enhanced computed tomography scan showed a heterogeneous pelvic-abdominal mass that measured 7.9 cm with uterine mass lesions (Fig. 1a). An exploratory laparoscopy was performed under the preoperative suspicion of intra-abdominal hemorrhage caused by torsion of a subserosal uterine fibroid. During laparoscopy, the subserosal uterine fibroid (11 cm in diameter) along with a large amount of hemorrhagic ascites was observed, as also shown on the preoperative computed tomography scan (Fig. 1b). The laparoscopy showed active bleeding from the superficial ruptured vessels overlying the subserosal fibroid on the uterine fundus (Fig. 2a). Detailed examination of the entire abdominal cavity did not reveal the torsion of fibroids and other areas of hemorrhage. The hemoperitoneum was thus diagnosed to be a result of spontaneous rupture of these vessels. The fibroid was enucleated and hemostasis of the bleeding site was attained with bipolar coagulation forceps (BiClamp; ERBE GmbH, Tubingen, Germany) (Fig. 2b, c). After the enucleation, the myometrial defect was closed in two layers using 0 PDS II sutures (Ethicon Japan, Tokyo, Japan) (Figs. 2d–f). The entire procedure of the laparoscopic surgery is presented in the video (Additional file 1). The surgery was completed in 100 min, and the total amount of blood lost was 400 mL. The specimen weighed 240 g. The postoperative course was uneventful, and the patient was discharged on the fourth day after the operation. Pathological examination confirmed the diagnosis of a benign uterine fibroid.

## Discussion and conclusions

In the current case, we successfully managed to diagnose spontaneous hemorrhage from the rupture of the superficial vessels overlying a uterine fibroid and subsequently treated the uterine fibroid by laparoscopic enucleation to remove the origin of the hemorrhage.

In literature, intra-abdominal hemorrhage due to uterine fibroids is rare with approximately 125 cases reported so far [11, 12]. As per a recent review, women who develop this complication typically present with hypovolemic shock and abdominal pain without a definite preoperative diagnosis, and the mortality rate is approximately 3.2% [10]. In most cases, bleeding from a uterine fibroid has been associated with trauma or torsion of pedunculated fibroids, while spontaneous rupture of the superficial vessels is extremely rare [13, 14]. In cases associated with bleeding, the source was mainly venous in origin [15]. There have been several hypotheses regarding the cause of spontaneous vascular rupture associated with uterine fibroid. One hypothesis is that the rupture of superficial vessels overlying the fiborid can be due to passive venous congestion associated with increased abdominal pressure during menstruation or when straining to pass stool, lifting heavy weights, or exercising [15–17]. Another hypothesis is that uterine fibroids greater than 10 cm in diameter might be associated with stretching and tension of the overlying vessels resulting in rupture [18]. Third hypothesis is that micro-RNAs, especially miR-29b which is a pivotal role to promote fibroid formation and upregulated mRNAs for multiple collagens in uterine fibroids [19], is recently reported to lead to the pathogenesis of leiomyoma [20], and progesterone downregulated miR-29b and upregulated mRNAs for multiple collagens in fibroids may induce to inhibit the growth of uterine fibroids [19]. In the current case, we speculate that the extreme congestion of the superficial veins of uterine fibroids owing to the withdrawal of progesterone during the late menstrual period along with the size of the uterine fibroid, which had a diameter of > 10 cm, may have contributed to the venous rupture. In addition, the decrement of progesterone on late menstrual period may contribute to upregulate miR-29b and downregulate mRNAs as the epigenetic change. and resulted in the rupture of the superficial vessels overlying a uterine fibroid.

A precise preoperative diagnosis is extremely difficult due to the rarity of this entity [12, 21]. Imaging techniques, such as ultrasound and computed tomography, are commonly used for preoperative examination, but in most cases, the preoperative diagnosis is unexplained hemoperitoneum. Recently, Scioscia and colleague [22, 23] commented that vascularity with doppler ultrasound may improve the detection rate of endometrial cancer which is relevant cause of abdominal uterine bleeding in pre- or perimenopausal women since vascularity the myometrium is not altered in fibroids, whereas it is aberrant in infiltrating endometrial cancer. In addition, Stabile et al. recommended to consider Meigs syndrome as the differential diagnosis on recognizing unexplained hemoperitoneum because they misdiagnosed an ovarian cancer due to the presence of a pelvic tumor, elevated CA-125 and ascites, and the patient underwent a total abdominal hysterectomy, salpingoophorectomy, removal of the pelvic mass, pelvic lymphadenectomy and peritoneal biopsies. peritoneal biopsies, even though it was Meigs syndrome with ovarian fibroma [24]. In this case, we regrettably misdiagnosed it as hemoperitoneum associated with the torsion of subserosal fibroids. Therefore, clinical examination with ultrasound, especially doppler ultrasound, is sufficient for preoperative diagnosis since surgery should not be delayed, especially in a setting of profound hemodynamic instability [25].

While supportive and resuscitative measures play a crucial role in the management of patients with massive intra-abdominal bleeding, surgeries such as hysterectomy and myomectomy should be performed immediately. The preferred procedure is hysterectomy for women who are postmenopausal and myomectomy for women of child-bearing age, since preserving the uterus should be a priority. If bleeding cannot be controlled, a hysterectomy must be considered [26]. In the current case, we chose the laparoscopic surgical approach for determining the origin of hemorrhage and subsequently performed hemostasis of the bleeding site and myomectomy with the repair of the uterine defect.

To our knowledge, this is the first report of a case in which a hemoperitoneum of ambiguous origin was laparoscopically diagnosed and treated by laparoscopic myomectomy for the removal of the origin of hemorrhage. However, a previous report has described the use of laparoscopy to diagnose the source of the bleeding and excision of the uterine fibroid by laparotomy [27].

In conclusion, although acute complications of uterine fibroids that require surgical intervention are exceptionally rare, hemorrhage from the rupture of the superficial vessels overlying a uterine fibroid should be included in the differential diagnosis of an unexplained hemoperitoneum. Surgeons should rapidly diagnose and manage women with acute abdominal pain and a history of uterine fibroids to prevent severe morbidity or even mortality. Based on our experience with this case, we recommend laparoscopic surgery in patients with stable hemodynamics.

## Supplementary Information

**Additional file 1.** The entire laparoscopic procedure followed for this case.

## Data Availability

The data that support the findings of this study are available from the corresponding author, S.F., upon reasonable request.

## Abbreviations

mRNAs: Messenger ribonucleic acids
CA-125: Cancer antigen-125
miR-29b: Micro ribonucleic acid-29b
